# Right retrocaval ureter associated with asymptomatic left retro aortic renal vein: A case report

**DOI:** 10.1016/j.ijscr.2025.111155

**Published:** 2025-03-14

**Authors:** Saida Hidouri, Sabrine Ben Ammar, Yosra El Mansouri, Fethi Jebali, Feten Letaief, Mohamed Ali Chaouch

**Affiliations:** a Research Laboratory LR12SP13, Faculty of Medicine of Monastir, Tunisia; b Department of Paediatric Surgery, Hospital of Zaghouan, Tunisia; c Department of Anesthesia-resuscitation B of Monastir, Tunisia; d Department of Anatomy, Faculty of Medicine of Monastir, Tunisia; e Department of Surgery, Medical School Hospital of Monastir, Tunisia

**Keywords:** Retrocaval ureter, Retro aortic renal vein, Uretero-nephrosis, Surgery, Child

## Abstract

**Introduction and importance:**

Retrocaval ureter is a rare congenital abnormality resulting from abnormal development of the inferior vena cava (IVC), leading to compression and obstruction of the right ureter. The condition is frequently diagnosed incidentally or presents symptoms related to hydronephrosis. The left retroaortic renal vein is another vascular anomaly with an estimated prevalence of 1.7 % to 3.4 %. However, it is typically asymptomatic.

**Case presentation:**

We present the case of a 9-year-old boy with a history of undescended right testis and hypospadias who presented with intermittent pain and hematuria. Physical examination revealed mild tenderness of the renal angle. Imaging studies, including ultrasound and uroscan, demonstrated right hydronephrosis, an S-shaped deformity of the proximal right ureter, and a medium calyceal stone. Interestingly, a left retroaortic renal vein was also identified. The patient underwent surgical correction using a conventional open approach, where the retrocaval segment was excised, excised, and an end-to-end uretero-ureteral anastomosis was performed on a double J stent. Postoperative recovery was uneventful and uneventful and follow-up imaging showed resolution of hydronephrosis.

**Clinical discussion:**

Retrocaval ureter is a developmental anomaly with an estimated prevalence of 0.13 %. It often presents urinary obstruction and recurrent infections. The diagnosis is based on imaging modalities, including 3D reconstruction CT urography. Treatment is recommended for symptomatic cases to prevent long-term renal deterioration. Surgical options include open, laparoscopic, and robotic approaches. In our case, conventional surgery was chosen due to the available anatomy and surgical expertise.

**Conclusion:**

Congenital anomalies of the IVC and renal veins, although rare, should be considered in paediatric patients with urinary compression. Advances in imaging have improved the diagnosis of these anomalies, allowing for timely and effective surgical management.

## Highlights

• Retrocaval ureter, a rare developmental defect of the inferior vena cava, can cause ureteral compression and hydronephrosis.
• A 9-year-old boy with a history of undescended testis and hypospadias presented with flank pain and hematuria; imaging revealed an "S"-shaped ureter and hydronephrosis.
• The patient underwent open surgical correction with ureteral anastomosis and a double-J stent, leading to full recovery.

## 1 Introduction

Abnormalities of the inferior vena cava (IVC) and renal veins are rare. However, with the increasing use of sectional imaging, these anomalies are more frequently diagnosed. The retrocaval ureter is a congenital malformation of the right ureter resulting from an unusual development of the IVC, and not of the urinary tract [1], the ideal nomenclature for the anomaly is preureteral vena cava, keeping in mind the aberrant embryology. The general prevalence of retrocaval ureter is estimated at 0.13 %, and this congenital abnormality seems to occur more often in men [2]. The prevalence of the left aortic retro renal vein is higher, it is estimated to be 1.7 % to 3.4 % [2]. The retrocaval ureter usually occurs during the 3rd to 4th decades of life, but may exhibit clinical symptoms in childhood [3]. Although this entity is rare, it has the potential to cause urinary obstruction with severe complications [4], hence the need for surgery in these symptomatic cases. However, for asymptomatic cases, simple observation is indicated.

We describe the case of symptomatic retrocaval, according to the SCARE guidelines [5], ureter in a 9-year-old boy with a surgical history of undescended palpated right testis and mid-shaft hypospadias; who was also incidentally diagnosed with a left renal vein.

## 2 Case presentation

**Fig. 1:**
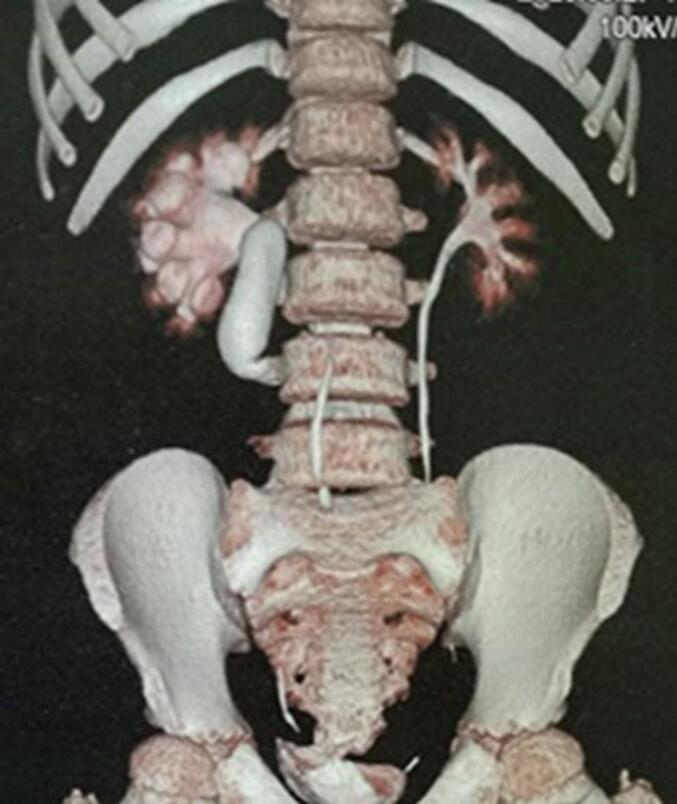
Uroscan shows right hydronephrosis and dilatation of the proximal ureter up to the level of L3 with reverse “J” deformity.

**Fig. 2:**
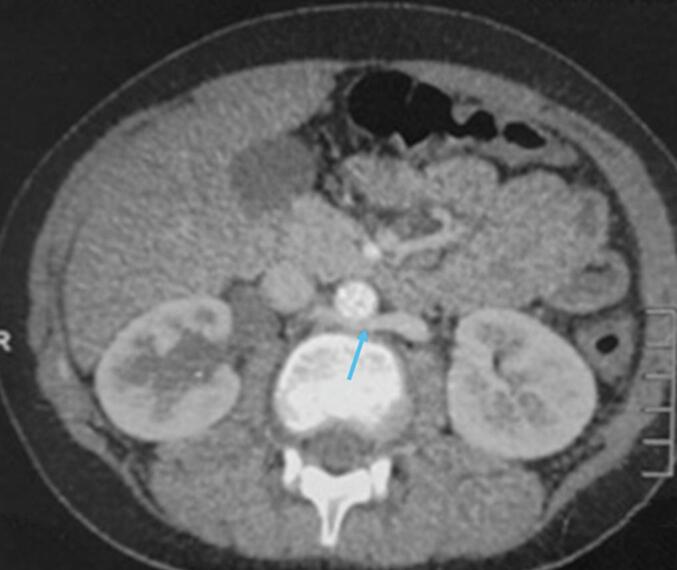
The Cross-section shows retroaortic left renal vein passing behind the abdominal aorta (blue arrow). (For interpretation of the references to colour in this figure legend, the reader is referred to the web version of this article.)

**Fig. 3:**
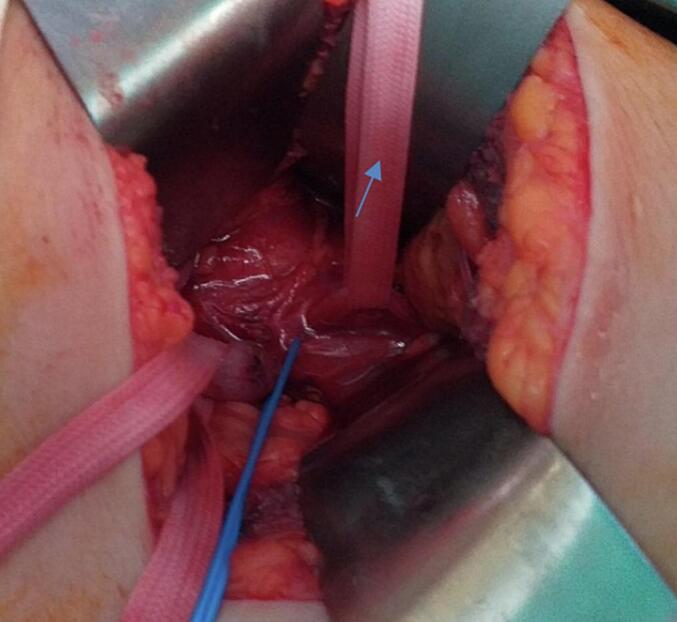
Intraoperative view of a right ureter (white surgical loop) with a dilated proximal segment, a normal distal one crossed by the inferior vena cava (blue surgical loop). (For interpretation of the references to colour in this figure legend, the reader is referred to the web version of this article.)

**Fig. 4:**
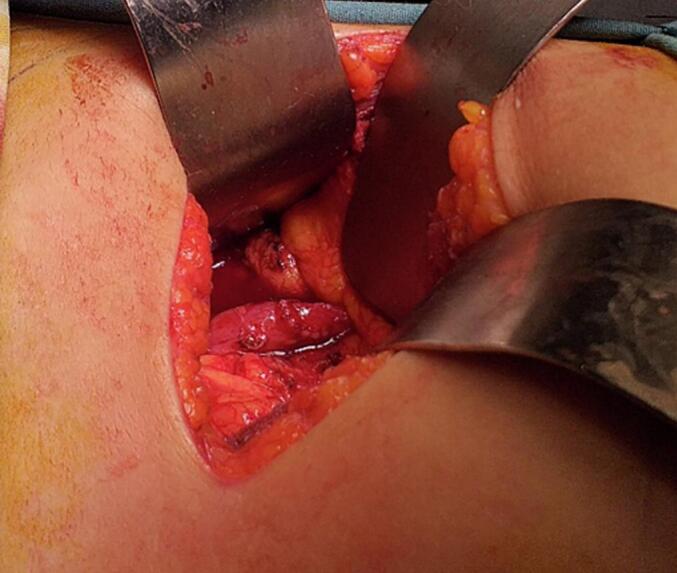
The final intraoperative view after the completed end-to-end ureteral anastomosis anterior to vena cava over a double-J stent.

It is about a 9-year-old patient who was operated on for an undescended palpated right testis at two years, then for mid-shaft hypospadias at three years of age. At that time, no investigation was conducted. Subsequently, he presented a 3-month history of paroxysmal moderate right flank colicky pain that suddenly appeared. When questioned, the patient also mentioned having hemorrhage. Physical examination revealed only mild tenderness at the renal angle tenderness. Biological data revealed hemoglobin 12 g/dL, blood urea nitrogen 3.32 mmol/L, and serum creatinine 39 μmol/L. Microscopic examination of the urine was normal. Abdominal ultrasound showed right hydronephrosis with a 12 mm pelvic stone. Uroscan revealed the right ureterohydronephrosis with an “S” shaped or reverse ‘J' deformity of the proximal ureter that abruptly **(**Fig. 1**)**, and 7 mm in the medium calyx. A left renal vein was also exhibited **(**Fig. 2**)**. The diagnosis of a symptomatic retrocaval ureter was made and the patient was operated on by conventional approach through an oblique incision in the right loin. The findings of the operation were that of the right retrocaval ureter, proximal dilated ureteral segment, and a normal distal segment located between the aorta and IVC **(**Fig. 3**)**. The redundant retrocaval segment was mobilized and excised, and end-to-end anastomosis was achieved on a double-J stent **(**Fig. 4**)**. During surgery, we did not find the stone. The postoperative course was uneventful. Histopathological examination of the excised segment showed normal ureteral stricture. The patient's symptoms resolved at follow-up and the double J stent was removed four weeks later. A regression of hydronephrosis was evident in ultrasound follow-up at 3-month.

## 3 Discussion

The development of the IVC is a complex process involving the formation and regression of three venous systems. Various classification systems have been proposed to categorize anomalies arising from abnormal IVC development. The most widely used classification is based on the affected segment of the final IVC. Among these anomalies, retrocaval ureter and retroaortic renal vein are notable vascular variations that can have clinical implications.

Retrocaval ureter, a rare congenital anomaly with an estimated prevalence of 0.13 %, occurs due to an abnormal persistence of the posterior cardinal vein, leading to ureteral entrapment behind the IVC [6]. Two types of retrocaval ureters have been described:

• Type I (Low Loop): The ureter crosses behind the IVC at the level of the third lumbar vertebra (L3), creating a characteristic reverse “J” or “S” shape. This is the most common type and was observed in our case.
• Type II (High Loop): A rarer variation where the ureter crosses the IVC at a higher level, near the renal hilum, often with a more horizontal orientation [12].

Our patient presented with a symptomatic Type I retrocaval ureter, which led to hydronephrosis and urolithiasis due to external compression. This is consistent with the literature, where lumbar pain, hematuria, and urinary tract infections are the most frequently reported symptoms [10]. The diagnosis was confirmed using CT urography, which demonstrated the classic S-shaped deformity, reinforcing the importance of advanced imaging in detecting and characterizing this condition [11]. In addition to the retrocaval ureter, our patient also had a left retroaortic renal vein (LRRV), another congenital vascular anomaly with an estimated prevalence of 1.7 % to 3.4 % [2]. The LRRV results from the persistence of an embryonic inter-subcardinal anastomosis, causing the left renal vein to pass posterior to the aorta before joining the IVC [7]. While usually asymptomatic, some cases have been associated with flank pain, hematuria, and left-sided varicocele due to compression (nutcracker effect) [9]. Unlike the retrocaval ureter, the LRRV in our patient was an incidental finding without clinical consequences. This underscores the variable clinical significance of congenital vascular anomalies, with some requiring surgical intervention while others remain benign. The presence of multiple anomalies in the same patient aligns with previous reports suggesting a potential association between IVC malformations and other urological or cardiovascular defects [8]. Advances in imaging, particularly 3D reconstruction CT urography and MR angiography, play a crucial role not only in diagnosing these anomalies but also in surgical planning. In our case, imaging helped delineate the course of the ureter and renal veins, guiding the choice of surgical approach and minimizing potential intraoperative complications [11]. Therapeutic options for retrocaval ureter range from conservative management in asymptomatic cases to surgical correction for those with obstructive symptoms. The standard approach involves ureteral anteriorization via excision of the retrocaval segment and end-to-end uretero-ureteral anastomosis, as performed in our case [13,14]. While laparoscopic and robotic approaches are gaining popularity, an open approach was chosen in our patient due to anatomical considerations and surgical expertise.

## 4 Conclusion

Even though anomalies of the IVC and renal veins are rare and often asymptomatic, surgeons should remain vigilant. Advances in imaging modalities have significantly improved the diagnosis and management of these conditions. High-resolution imaging techniques, such as CT urography with 3D reconstruction and MR urography, not only aid in accurately identifying vascular and ureteral anomalies but also facilitate preoperative planning. By providing a detailed anatomical roadmap, these imaging tools help surgeons select the most appropriate surgical approach, reducing complications and optimizing patient outcomes.

## Authors' contributions

All authors participated in the treatment of the patients, writing, and approving the manuscript.

## Consent

Written informed consent was obtained from the patient's parents/legal guardian for publication and any accompanying images. A copy of the written consent is available for review by the Editor-in-Chief of this journal on request.

## Patient consent

Written informed consent was obtained from the patient to publish this case report and accompanying images. On request, a copy of the written consent is available for review by the Editor-in-Chief of this journal.

## Ethical approval

Ethical approval is exempted/waived at our institution.

## Guarantor

Mohamed Ali Chaouch.

## Funding

This research did not receive specific grants from the public, commercial, or nonprofit sectors.

## Declaration of competing interest

No conflict of interest to disclose.
